# Brain activity classifies adolescents with and without a familial history of substance use disorders

**DOI:** 10.3389/fnhum.2015.00219

**Published:** 2015-04-22

**Authors:** Jianping Qiao, Zhishun Wang, Lupo Geronazzo-Alman, Lawrence Amsel, Cristiane Duarte, Seonjoo Lee, George Musa, Jun Long, Xiaofu He, Thao Doan, Joy Hirsch, Christina W. Hoven

**Affiliations:** ^1^Department of Electronics, College of Physics and Electronics, Shandong Normal UniversityJinan, China; ^2^Department of Psychiatry, Columbia University and The New York State Psychiatric InstituteNew York, NY, USA; ^3^Department of Computer Engineering, School of Information Science and Engineering, Central South UniversityChangsha, China; ^4^Departments of Psychiatry and Neurobiology, Yale School of MedicineNew Haven, CT, USA; ^5^Department of Epidemiology, Mailman School of Public Health, Columbia UniversityNew York, NY, USA

**Keywords:** substance use disorders, family history, risk, fMRI, emotional conflict, brain connectivity, machine learning

## Abstract

We aimed to uncover differences in brain circuits of adolescents with parental positive or negative histories of substance use disorders (SUD), when performing a task that elicits emotional conflict, testing whether the brain circuits could serve as endophenotype markers to distinguish these adolescents. We acquired functional magnetic resonance imaging data from 11 adolescents with a positive familial history of SUD (FH+ group) and seven adolescents with a negative familial history of SUD (FH− group) when performing an emotional stroop task. We extracted brain features from the conflict-related contrast images in group level analyses and granger causality indices (GCIs) that measure the causal interactions among regions. Support vector machine (SVM) was applied to classify the FH+ and FH− adolescents. Adolescents with FH+ showed greater activity and weaker connectivity related to emotional conflict, decision making and reward system including anterior cingulate cortex (ACC), prefrontal cortex (PFC), and ventral tegmental area (VTA). High classification accuracies were achieved with leave-one-out cross validation (89.75% for the maximum conflict, 96.71% when combining maximum conflict and general conflict contrast, 97.28% when combining activity of the two contrasts and GCIs). Individual contributions of the brain features to the classification were further investigated, indicating that activation in PFC, ACC, VTA and effective connectivity from PFC to ACC play the most important roles. We concluded that fundamental differences of neural substrates underlying cognitive behaviors of adolescents with parental positive or negative histories of SUD provide new insight into potential neurobiological mechanisms contributing to the elevated risk of FH+ individuals for developing SUD.

## Introduction

Substance Use Disorder (SUD) refers to pathological use and/or dependence on a drug or other chemicals leading to detrimental effects to the individual's physical or mental health and the welfare of others (Pitkanen et al., 2008). SUD is highly transmitted across generations (Schuckit et al., 2000, Clark et al., 2004). It is well-established that individuals with a positive family history (FH+) of SUD are at a significantly increased risk for problems with emotional processing (Oscar-Berman and Bowirrat, 2005) and associated disruptions in executive functioning (Sinha et al., 1989), which could, in turn, increase risk for SUD (Fox et al., 2007, 2008; Cservenka et al., 2014b). Therefore, it would be important to identify the mechanism of familial history risk by examining adolescents at high risk for SUD, while still substance naïve, to capture putative factors through behavioral and neuroimaging methods.

Deficits in processing emotion and in controlling impulsivity that are frequently observed in SUD individuals suggest the abnormalities of neural networks that regulate emotion (Montagne et al., 2006; Foisy et al., 2007; Salloum et al., 2007). Functional imaging studies have revealed deficits of neural activity in cognitive control and emotion regulation as well as reward system in SUD individuals (O'daly et al., 2012; Muller-Oehring et al., 2013). As the high risk of mental disorders of SUD family, several brain areas associated with emotional and reward dysfunction are affected in FH+ individuals including prefrontal gyrus, insula, putamen (Heitzeg et al., 2008), amygdala (Glahn et al., 2007), and nucleus accumbens (NAcc) (Andrews et al., 2011) compared to negative family history (FH−) individuals during various tasks, such as a monetary incentive delay (MID) task (Andrews et al., 2011), a task of viewing words with positive, negative, or neutral valence (Heitzeg et al., 2008) and a recognition task using faces expressing fear vs. geometric objects (Glahn et al., 2007). Structural imaging studies have also revealed reduced amygdala volumes in FH+ subjects compared to age-matched controls (Hill et al., 2001). A key limitation of these studies that investigated the neural bases of high risk of SUD was that they mainly focused on adults or late adolescence. Generally, SUD initiation begins in early adolescence with rates of SUD increasing sharply between ages 12 and 21 (Wu et al., 2008; Volkow et al., 2010). Therefore, it is critical to examine neural mechanism that may distinguish FH+ from FH− among early-middle adolescence. According to one of the few studies that focused on this age group, FH+ adolescents showed less inhibitory frontal response than FH− adolescents when performing a go/no-go task (Schweinsburg et al., 2004). Recently, another investigation found that FH+ individuals showed less activity than FH− individuals in the prefrontal gyrus during a spatial working memory task than vigilance condition (Mackiewicz Seghete et al., 2013) while showed greater activation in frontal-limbic search territory during a stroop color naming task (Silveri et al., 2011). Another study examined intrinsic functional connectivity with fMRI and showed differences involving the NAcc in the reward circuit between FH+ adolescents and FH− adolescents (Cservenka et al., 2014a).

Despite these few studies focused on cerebral activation of FH+ families during early or middle adolescence, the brain circuitry that might govern the aggregation of SUD in families remains unclear. Moreover, most of the previous studies focus on the reward or memory system such as the MID or spatial working memory task. To date, investigations have not reported on circuit-based neural mechanisms that might govern the high risk for developing SUD, based on findings from an emotion-related task such as an emotional stroop.

Therefore, to characterize the altered processing of emotional and cognitive controls in the brains of FH+ adolescents, an fMRI study was conducted on FH+ adolescents and age-matched FH− adolescents during an emotional stroop task in this study. This emotional stroop task aimed to examine the emotional regulation and cognitive control processing which included a total of four conditions based on combinations of two current trial types (congruent and incongruent) and two preceding trial types (congruent and incongruent) (Etkin et al., 2010). Among the four conditions, the condition with the current incongruent trials preceded by congruent trials had maximal interference, whereas the condition with the current congruent trials preceded by congruent trials had least interference. Because the contrast between the two conditions could potentially induce a maximal conflict for the subjects, it was chosen as the focus of the present analysis, which has the goal of detecting conflict-related activity in the fMRI data. In addition, neural activity from the condition reflecting a general conflict, i.e., all incongruent trials vs. all congruent trials regardless of the previous trial types was also investigated (Kerns et al., 2004). We then performed a second-level analyses on contrast images to detect random effect of activity within and between the FH+ and FH− groups. Furthermore, the granger causality index (GCI) between brain regions showing significant group differences were calculated to investigate how these regions interacted.

In general, the ultimate goal of fMRI studies is to uncover neural circuits that govern human behaviors and psychiatric disorders, thereby identifying biomarkers that can robustly predict specific behaviors or disorders. Many studies have demonstrated that machine learning and pattern classification techniques are useful for identifying potential biomarkers for the diagnosis of psychological disorders and understanding their etiology (Nieuwenhuis et al., 2012; Westman et al., 2012; Gray et al., 2013). To our knowledge, however, no study has utilized this methodology to successfully classify adolescence as FH+ or FH− based on brain activity and connectivity. Different tasks examine different brain function and its related neural networks. We aimed to detect abnormalities associated with emotional dysfunction and cognitive control by using emotional stroop task. The features identified in this task will be different and irreplaceable by that of other tasks such as MID. Therefore, we performed a machine learning procedure on the adolescent's fMRI data to classify them as either FH+ or FH− for SUD. The FH+ individuals may have deficits in conflict adaptation and cognitive control which may result a high risk of SUD. This method was expected to help cross-verify whether the detected abnormities in brain activity and connectivity could serve as neuromarkers for SUD risk, if they could objectively distinguish the FH+ group from the FH− group.

We hypothesized that FH+ adolescents would show hyperactivity in the regions related to emotional conflict regulation and cognitive control in response to the emotional stroop task stimuli. We expected that brain activity and connectivity with significant differences could serve as features for classification and prediction between FH+ and FH− adolescents.

In this study, all the families come from impoverished, high-crime neighborhoods of the South Bronx, NYC; thus, equally exposed to neighborhood stressors. In the adults, we only measured depressions and anti-social behavior, and those who were screened positive were excluded from the fMRI study. Most of the index parents were also involved with the criminal justice system. Families were excluded if the child had any DSM diagnosis. Given that they are all from high-crime, impoverished neighborhoods, and the index parents have criminal justice system involvement, makes them at high-risk for SUD. One limitation of the study is that we do not consider environmental contribution to the development of SUD as substance use has significant environmental risk factors.

## Materials and methods

### Participants

Eighteen well-characterized parents and their children, participants in a large, longitudinal epidemiological investigation in New York City, were recruited for this study which included 11 FH+ adolescents (seven males, four females, 12.7 ± 1.3 years old) and seven FH− adolescents (four males, three females, 13.6 ± 1.3 years old) (Table 1). The household income and criminal justice system of the FH+ and FH− parents are shown in Table 2. The parents are negative for depression and antisocial behavior (excluded from the study). The participants come from mostly low to lower-middle income families and are all minority (10 Black/African-American (not Hispanic), five Hispanic and three Other/Mixed (not Hispanic), living in high-risk, disadvantaged neighborhoods of the South Bronx, New York City. Thirteen (72.2%) of the children/adolescents received mostly A's or B's in their last report card, while five (27.8%) received mostly C's or D's. Eight (72.7%) of the 11 FH+ children had a SUD+ father and three (27.3%) had a SUD+ mother; three of the mothers reported smoking and one reported drinking alcohol while pregnant with their child. While four (22.2%) of the participating children have asthma, no other chronic physical health problems were reported, and none of the children/adolescents were on medication although one child reported occasionally using an asthma pump when necessary.

**Table 1:** **The demographic characteristics of FH+ and FH− adolescents**.

	**FH+ adolescents (*N* = 11)**	**FH− adolescents (*N* = 7)**	***P***
Female, *n* (%)	4 (36.4)	3 (42.9)	0.7829
Race/Ethnicity, *n* (%)			
Hispanic	4 (36.4)	1 (14.3)	0.4211
Black (non-Hispanic)	6 (54.5)	4 (57.1)	
Mixed/Other (non-Hispanic)	1 (9.0)	2 (28.6)	
Age, mean (SD)	12.7 (1.3)	13.6 (1.3)	0.1889

**Table 2:** **The demographic characteristics of FH+ and FH− parents**.

	**FH+ parents (*N* = 11)**	**FH− parents (*N* = 7)**	***p***
Age, mean (SD)	44.7 (6.4)	45.9 (7.7)	0.7401
Female, *n* (%)	4 (36.4)	2 (28.6)	0.7324
Race/Ethnicity, *n* (%)			
Hispanic	3 (27.3)	1 (14.3)	0.7251
Black (non-Hispanic)	5 (45.4)	3 (42.9)	
Mixed/Other (non-Hispanic)	3 (27.3)	3 (42.9)	
Household income^*^, *n* (%)			
Less than $1500	6 (60.0)	1 (14.3)	0.1693
$15,000–$50,000	2 (20.0)	3 (42.9)	
More than $50,000	2 (20.0)	3 (42.9)	
Criminal justice system involvement, *n* (%)	10 (90.9)	3 (42.9)	**0.0265**

* *One-family with missing data*.

*The bold indicates that the comparisons are significant (p < 0.05, uncorrected)*.

All participants provided a urine sample at the time of the fMRI. A safety screening form, Edinburgh Handedness Inventory, and informed consent form were approved by the Columbia University-New York State Psychiatric Institute Institutional Review Board. Consent or assent forms were obtained from all participants and parental consent was obtained from participants under age 18. Only right-handed participants were recruited to reduce heterogeneity with respect to laterality of brain functions of interest.

### Experimental design

**Figure 1:**
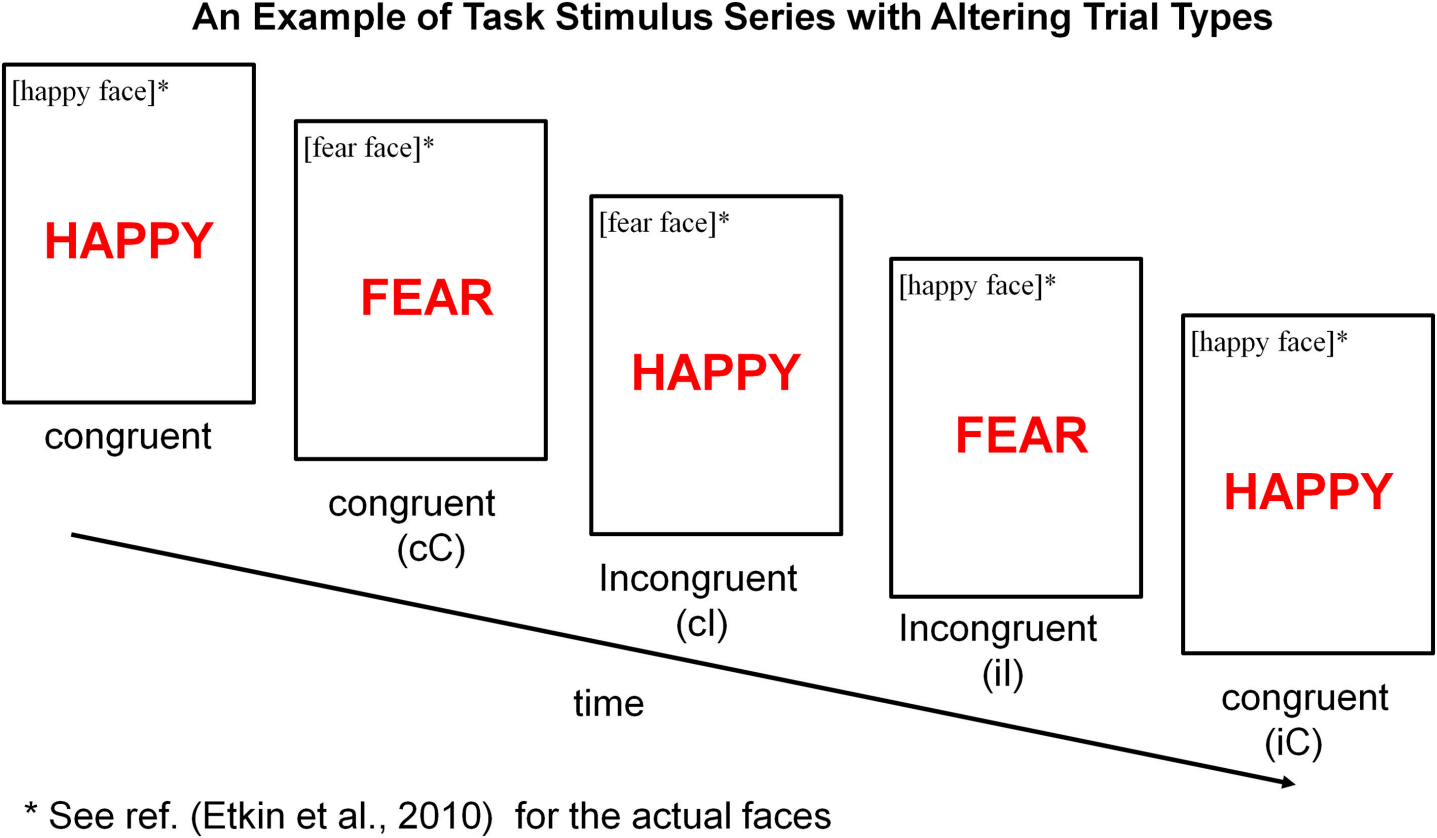
**Emotional stroop task**. Participants were instructed to identify the underlying facial emotion (fear or happy) while ignoring an overlying emotion word (“FEAR” or “HAPPY”). Trials varied such that emotional distracter words either matched [“congruent” (C)] or conflicted [“incongruent” (I)] with the underlying facial expression. Depending on a combination of current trial type (C or I) and preceding trial type (denoted using a lower case letter, c or i), there were four joint trial types for any current trial when considering its preceding trial type: cC (post-congruent congruent), cI (post-congruent incongruent), iI (post-incongruent incongruent) and iC (post-incongruent congruent). Maximum Conflict was isolated by contrasting cI trials with cC trials. General Conflict interference was assessed by contrasting cI or iI trials with cC or iC trials.

Each participant performed an emotional stroop task (see Figure 1) (Etkin et al., 2010) during fMRI scan. The task consisted of 148 presentations of happy or fearful facial expression photographs drawn from Ekman and Friesen (1976). Faces were cropped and the words “FEAR” or “HAPPY” were written in prominent red letters across the face. Thus, stimuli were congruent (C) or incongruent (I) with respect to facial expression and word, which created emotional conflict. In other words, emotional conflict arose from incompatibility between the task-relevant and task-irrelevant emotional dimensions of a stimulus. Stimuli were presented for 1 s, with a varying inter-stimulus interval (ISI) of 3–5 s (mean ISI = 4 s) during which a central fixation cross was shown. Stimuli were presented in a pseudorandom order (counterbalanced for equal numbers of C–C, C–I, I–C, I–I stimulus pairings). Facial identities, expressions and genders were randomized throughout the task, and stimulus occurrences were counterbalanced across trial types and response buttons. Subjects were instructed to identify the emotional expression of faces and to respond as fast and accurately as possible, by pushing response buttons corresponding to “FEAR” (right index finger) or “HAPPY” (right middle finger) for the emotion expressed by the faces.

### Image acquisition

Imaging was performed using a Signa 1.5 Tesla (GE Medical Systems, Milwaukee, Wisconsin) MRI scanner at the Functional MRI Research Center, Neurological Institute, Columbia University. During fMRI scanning, stimuli were presented with presentation software and displayed on VisuaStim XGA LCD screen goggles (Resonance Technology, Northridge, CA). Functional data were acquired using a gradient-echo, T2^*^-weighted echoplanar imaging (EPI) with blood oxygen level-dependent (BOLD) contrast pulse sequence. Twenty-four contiguous axial slices were acquired along the AC-PC plane, with a 64 × 64 matrix and 20 cm field of view (voxel size = 3.125 × 3.125 × 4.0 mm, repetition time = 2000 ms, echo time = 40 ms, flip angle = 60°).

### Image analysis

Functional MRI data were analyzed using SPM8 (Welcome Department of Imaging Neuroscience, London, UK) implemented in MATLAB 2012B. During the preprocessing, the functional volumes were slice timing-corrected, spatially realigned to correct for motion, normalized to Montreal Neurological Institute (MNI) coordinate system (Friston et al., 1995), resampled at 3 × 3 × 3 mm^3^, and spatially smoothed with an isotropic 8 mm full-width at half-maximum Gaussian kernel to remove spatial noise and compensate for residual variability in functional anatomy after spatial normalization, as well as to facilitate application of Gaussian random field theory for adjusted statistical inference. The spatially smoothed functional data were high-pass filtered via a discrete cosine transform at a cutoff frequency of 1/128 Hz to remove low-frequency noise such as scanner drift.

There were a total of four conditions based on the combinations of the two current trial types (congruent and incongruent) and the two preceding trial types (congruent and incongruent). Only correct trials were included in the conditions and the error trials were modeled separately. The four conditions were named cC, iC, cI, iI, where capital letters C and I indicated that the current trials were congruent and incongruent, respectively; whereas lower case letters c and i indicated that the preceding trials were congruent and incongruent, respectively. We analyzed the functional image data using general linear model implemented in SPM8 at two levels: the individual level (first-level) to detect task-related (conflict-related) activity within each individual participant; the group level (second-level) to detect random effect of task-related activity within and between diagnostic groups.

In the first-level analysis (Friston et al., 1994) we modeled the functional data for each participant with four independent functions, each of which was generated by convolving a hemodynamic response function with a boxcar function derived from the onsets and durations of each of the four conditions, cC, iC, cI, iI. Therefore, five regressors were created including cC, iC, cI, iI and an error term. To adjust confounding effects of subject movement, six motion parameters were also included. We estimated the model using the restricted maximum likelihood algorithm with an option of serial correlation removal via the first-order autoregressive model and then generated *T* contrast images, including cI vs. cC and I vs. C (cI + iI vs. iC + cC), which reflected the maximal conflict and general conflict, respectively under the emotional stroop task.

The contrast images generated by the first-level models were used to group-level random-effect analysis. We inputted cI vs. cC and I vs. C contrast images from each subject into the second-level two-sample *t*-test model which was implemented in SPM8 factorial module to detect a random effect of the group difference of the conflict-related activity between the FH+ and FH− adolescents. Then time courses of the brain regions with significant neural activity in the three contrasts were extracted to compute granger causality indices (GCIs) (Granger, 1969; Chen et al., 2004) to assess causal interactions.

### Feature extraction and classification

The following brain features were selected for classification: (1) eigenvectors that were extracted from contrast images within the activated regions. We detected brain regions with significant group differences after second-level analysis. The values of these regions in the contrast images were defined as the first part of feature vector. (2) GCIs that had significant differences between FH+ and FH− were defined as the second part of feature vector. The dimension of feature vector for each subject is N^*^1 in which *N* is the number of the detected regions and GCIs. In this study, several regions or GCIs with the most significantly differences were selected, so *N* was not much larger than the training sample size which eliminated over-fitting problem in the classification.

In pattern recognition, many different algorithms have been developed for classification such as hidden Markov models, support vector machine (SVM), and neural network. SVM is a universal learning machine proposed by Vapnik (1995), which has been widely used in the classification of psychological disorders and normal controls such as schizophrenia (Nieuwenhuis et al., 2012), Alzheimer's disease (Gray et al., 2013) and depression (Gong et al., 2011). In this study, we used SVM to classify the status of family (parental) history of SUD and thus to discriminate between FH+ and FH− adolescents. Details of the SVM algorithm are shown in the Supplementary Material. The support vector optimization problem can be solved analytically only when the number of training data is very small (Burges, 1998). In addition, five-fold cross validation (CV) and leave-one-out cross validation method were used to quantify the performance of SVM classifier which gives an estimate of how well the model will generalize to a new data set. We combined two common approaches for reducing optimism between in-sample correlation model and out-of-sample generalizable model, cross-validation and randomly split data into training and testing sets (Gabrieli et al., 2015). In detail, the training and testing set were selected randomly according to different CV methods. In five-fold CV, four-folds were used for training and the last fold was used for testing. The process was repeated 1000 times to determine a null-distribution of accuracies. In leave-one-out CV, one of the observations was randomly selected, others were employed for training. The procedure was also repeated for 1000 times. Each classification accuracy was calculated by averaging 1000 CV trials with randomly selected subsets. Classification performance was compared between different cross-validation methods. Therefore, the sample size in this study is not large but remains acceptable for binary classification. Importantly, the accurate classification was able to cross verify the features that detected by brain activity and connectivity and identify the difference between FH+ and FH− individuals.

The permutation test is a non-parametric technique in which a reference distribution is obtained by calculating all possible values of the test statistic under rearrangements of the labels of the samples (Golland and Fischl, 2003). We additionally performed permutation tests to obtain the *p*-value of the classification accuracies, sensitivity and specificity, reflecting how certain we are that the result is not due to chance (Schrouff et al., 2013).

## Results

### Behavioral results

**Figure 2:**
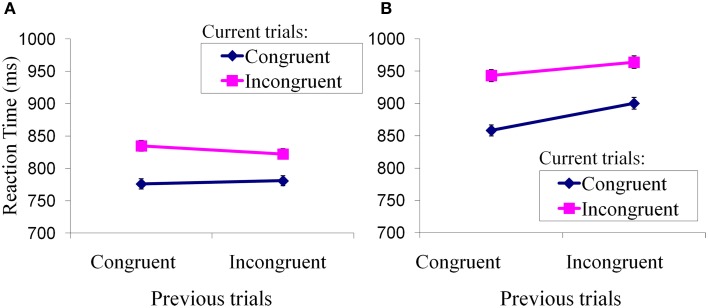
**Performance of Emotional Stroop Task**. Mean reaction times (± standard errors) of current trial congruency were plotted under modulation of preceding trial congruency. **(A)** Adaptation to conflicts in FH− children; **(B)** Adaptation to conflicts in FH+ children.

We performed a repeated-measures analysis of variance with the factors of trial type (incongruent, congruent) and group (FH+, FH−) on the reaction time (RT) for each trial type across the 11 FH+ adolescents and seven FH− adolescents (Figure 2). FH− adolescents showed less reaction time for iI than cI trial (Figure 2A). In contrast, FH+ adolescents showed greater reaction time for iI than cI trial (Figure 2B). Thus, the FH− adolescents had a typical reaction time pattern consistent with the literature (Kerns et al., 2004) that has addressed this type of conflict-related task, while FH+ adolescents did not have this pattern. This indicates that FH+ adolescents failed to adapt to the incongruent conflict. *Post-hoc* analyses further indicated that mean reaction times of FH+ adolescents were generally greater than those of FH− adolescents during the performance of each of the four trial types (cC: 858.30 ± 67.05 vs. 776.01 ± 10.96; iC: 900.18 ± 67.44 vs. 781.04 ± 16.10; cI: 943.12 ± 68.67 vs. 834.80 ± 15.59; iI: 963.91 ± 78.11 vs. 822.33 ± 23.73; incongruent: 953.52 ± 72.56 vs. 828.56 ± 17.74; congruent: 879.24 ± 67.14 vs. 778.53 ± 13.08). We also conducted two-tailed two-sample *t-test* comparing the reaction time of FH+ individuals to that of FH− individuals for each trial. A probability of *p* smaller than 0.05 was considered significant to reject the null hypothesis that the means are equal. No trial differed significantly. This indicates that FH+ adolescents took a longer time, in general, to perform an emotional stroop task than did FH− adolescents.

### Image results

#### Maximum conflict (cI vs. cC)

**Figure 3:**
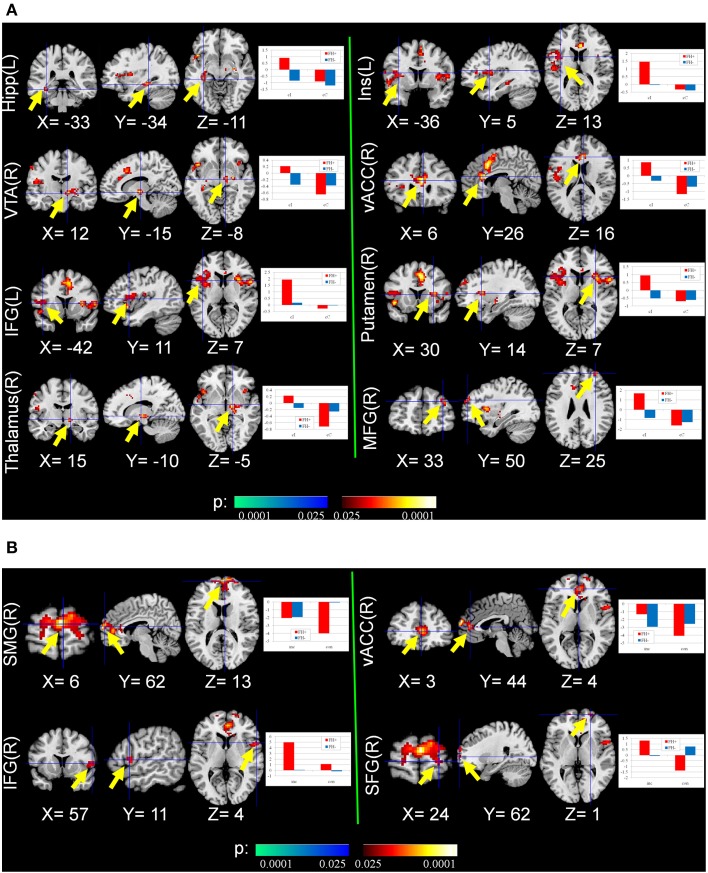
**Group differences of conflict-related activity between the FH+ and FH− children. (A)** congruent and incongruent trials vs. congruent and congruent trials (cI_cC), detecting the activity in response to maximal conflicts in the task; **(B)** incongruent trials vs. congruent trials (I_C), detecting the activity in response to general conflicts in the task. Hipp, Hippocampus; Ins, Insula; VTA, ventral tegmental area; vACC, ventral anterior cingulate cortex; IFG, inferior frontal gyrus; MFG, middle frontal gyrus; SMG, superior medial gyrus; SFG, superior frontal gyrus.

From the normal behavior pattern as shown in Figure 2A, the reaction time difference between cI (most difficult and slowest) and cC (easiest and fastest) is the maximum and thus reflects maximal conflict. Therefore, the contrast cI_cC was defined as one of the measures of fMRI. Significant differences were found in the second-level analysis on the contrast images cI vs. cC in the regions related to conflict, decision making and reward system [*p* = 0.01, corrected by cluster extent thresholding with *k* = 30, which was determined by Monte Carlo Simulation (Slotnick et al., 2003)], including anterior cingulate cortex (ACC), insula, prefrontal cortex (PFC), middle cingulate cortex (MCC), ventral tegmental area (VTA), superior temporal gyrus (STG), putamen, precentral gyrus, thalamus, amygdala, and hippocampus (Figure 3A, Table 3). The PFC and ACC are related to decision making. The VTA has been shown to be related to drug addiction (Zijlstra et al., 2009).

**Table 3:** **The locations of activations for the group difference between FH+ and FH− adolescents**.

**Activated area**	**Location side**	**Peak location**			***T* statistic**	**Classification contribution**
		***x***	***y***	***z***		
**cI_cC EFFECTS: FH+ VS. FH− (POSITIVE)**						
Hippocampus (Hipp)	L	−33	−34	−11	+3.99	0.4617
Insula	L	−36	5	13	+3.28	0.1443
	R	45	8	−2	+3.20	0.4265
Superior temporal gyrus (STG)	L	−54	5	−5	+3.22	0.4183
Inferior frontal gyrus (IFG)	L	−42	11	7	+3.02	0.1856
Putamen	R	30	14	7	+4.02	0.1263
Thalamus	R	15	−10	−5	+4.37	0.2998
Amygdala	R	27	−13	−8	+3.86	0.2212
Ventral anterior cingulate cortex (vACC)	R	6	26	16	+4.61	0.4758
Middle cingulate cortex (MCC)	R	6	17	40	+4.55	0.3718
Ventral tegmental area (VTA)	R	12	−15	−8	+3.54	0.3764
Precentral Gyrus	L	−48	−4	46	+3.47	0.1979
Middle frontal gyrus (MFG)	R	33	50	25	+3.67	0.1251
**I_C EFFECTS: FH+ VS. FH− (POSITIVE)**						
Superior medial gyrus (SMG)	R	6	62	13	+5.60	0.7273
Ventral anterior cingulate cortex (vACC)	R	3	44	4	+4.54	0.7542
Superior frontal gyrus (SFG)	L	−15	59	22	+3.21	0.5096
	R	24	62	1	+3.21	0.1447
Inferior frontal gyrus (IFG)	R	57	11	4	+3.51	0.7616

*NA, Not applicable*.

*All coordinates are in the Montreal Neurological Institute ICBM 152 template*.

#### General conflict (I vs. C)

Neural activity related to I vs. C contrast reflecting general conflict was also examined. Compared with FH− adolescents, FH+ adolescents activated brain regions including PFC and ACC more strongly to resolve general conflict [*p* = 0.01, corrected by cluster extent thresholding with *k* = 30, which was determined by Monte Carlo Simulation (Slotnick et al., 2003)] (Figure 3B, Table 3).

#### Granger causality interactions

Two types of GCIs were computed between the time courses of the activated regions in Table 3 for each participant. One showed influence of region A on region B, the other showed influence of region A on region B through the thalamus. This is possibly due to the top-down circuit from cortex to basal ganglia being direct, while the bottom-up circuit from basal ganglia to cortex is via the thalamus. We conducted two-tailed two-sample *t*-test comparing the GCIs of FH+ individuals to that of FH− individuals. A probability of *p* smaller than 0.05 was considered significant to reject the null hypothesis that the means are equal. The FH+ group showed weaker causal influences compared with the FH− group regarding the connections from inferior frontal gyrus (IFG) to ACC (0.0503 ± 0.0657 vs. 0.2333 ± 0.1325, *t* = −3.9261, *p* = 0.0012), from insula to ACC (0.1157 ± 0.1100 vs. 0.2671 ± 0.1112, *t* = −2.8369, *p* = 0.0119). No significant differences from PFC [including Superior Medial Gyrus (SMG), Superior Frontal Gyrus (SFG), Middle Frontal Gyrus (MFG), IFG] to VTA, from VTA to PFC via thalamus were observed (Table 4).

**Table 4:** **Brain connectivity (granger causality indices, GCIs) for the group difference between FH+ and FH− adolescents**.

	**GCIs of FH+ group (mean ± std)**	**GCIs of FH− group (mean ± std)**	**GCIs of FH+ group vs. FH− group**	**Classification contribution**
MFG → SMG	0.06 ± 0.04 (*p* = 1.7801 e-04)	0.17 ± 0.05 (*p* = 1.5438 e-04)	***t* = −4.95, *p* = 0.0001**	0.0565
IFG → SMG	0.07 ± 0.06 (*p* = 0.0018)	0.19 ± 0.09 (*p* = 0.0013)	***t* = −3.39, *p* = 0.0038**	0.0714
Insula → ACC	0.12 ± 0.11 (*p* = 0.0058)	0.27 ± 0.11 (*p* = 7.0975 e-04)	***t* = −2.84, *p* =** **0.0119**	0.1129
IFG → ACC	0.05 ± 0.07 (*p* = 0.0294)	0.23 ± 0.13 (*p* = 0.0035)	***t* = −3.93, *p* = 0.0012**	0.1375
IFG → Hippo	0.04 ± 0.03 (*p* = 2.8150 e-04)	0.05 ± 0.03 (*p* = 0.0080)	*t* = −0.35, *p* = 0.7284	NA
IFG → Putamen	0.06 ± 0.04 (*p* = 0.0016)	0.08 ± 0.07 (*p* = 0.0337)	*t* = −0.83, *p* = 0.4214	NA
IFG → VTA	0.08 ± 0.04 (*p* = 6.982 3e-05)	0.10 ± 0.06 (*p* = 0.0055)	*t* = −0.71, *p* = 0.4880	NA
Thalamus Putamen → IFG	0.38± 0.21 (*p* = 0.0027)	0.44 ± 0.22 (*p* = 4.1792 e-04)	*t* = −0.58, *p* = 0.5705	NA
Thalamus VTA → IFG	0.59 ± 0.18 (*p* = 0.0149)	0.62 ± 0.19 (*p* = 0.0037)	*t* = −0.41, *p* = 0.6889	NA

*The data in each cell in the second and third column represent the mean ± std of the Granger causality index. We used two-sample t-test to compare the GCIs between FH+ and FH− groups. The bold in the fourth column indicates that the comparisons are significant (p < 0.05, uncorrected). Thalamus X → Y represents the connectivity between X and Y via the thalamus. NA in the last column means that the GCIs were not used as features for classification*.

### Feature selection and classification results

Two cross-validation (CV) methods were applied, including five-fold CV and leave-one-out CV in four sets of features for SVM. These are features of the general conflict contrast (I_C), maximum conflict contrast (cI_cC) features from combining the two contrasts (I_C and cI_cC), and multiple type features from combining activity of the two contrasts and effective connectivity GCIs with significant group differences (I_C and cI_cC and GCIs). Take cI vs. cC contrast as an example, we inputted cI vs. cC contrast images from each subject into the second-level two-sample *t*-test model. We detected the brain regions with significant differences and then extracted the values of these regions in cI vs. cC contrast image as features for each subject. The brain regions of the contrast images that showed significant differences in the results of the two-sample two-tailed *t*-tests between the FH+ and FH− groups were used as features (Dai et al., 2012). Using the activated featured brain areas resulting from the I_C, cI_cC and GCIs, we performed SVM to classify the FH+ and FH− groups, yielding an average accuracy of 96.60% (the corresponding *p*-value that was obtained using a permutation test with 1000 repetitions for the classifier was 0.002) with the five-fold CV method and 97.28% (the corresponding *p*-value that was obtained using a permutation test with 1000 repetitions for the classifier was 0.003) with the leave-one-out CV method. Classification performance was compared between different CV methods and feature sets. Higher classification accuracy was obtained by combining different sets of features (Table 5).

**Table 5:** **Classification results of support vector machine (SVM)**.

**Feature sets**	**Average accuracy % (*p*-value)**	
	**SVM with five-fold CV**	**SVM with leave-one-out CV**
Features from maximum conflict contrast (cI_cC)	87.39% (0.01) (std = 0.0015)	89.75% (0.01) (std = 0.0076)
Features from general conflict contrast (I_C)	91.71% (0.01) (std = 0.0011)	92.34% (0.01) (std = 0.0069)
Features from combining the maximum conflict contrast and general conflict contrast (I_C and cI_cC)	94.35% (0.008) (std = 0.0009)	96.71% (0.008) (std = 0.0066)
Multiple type features from combining activity of two contrasts and connectivity GCIs (I_C and cI_cC and GCIs)	96.60% (0.002) (std = 0.0008)	97.28% (0.003) (std = 0.0047)

*The training and testing set were selected randomly according to different CV methods. In the five-fold CV, four-folds were used for training and the last fold was used for testing. This process was repeated five times, leaving one different fold for evaluation each time. In the leave-one-out CV, one of the observations was randomly selected, others were employed for training. The procedure was repeated for 1000 times. Each classification accuracy was calculated by averaging 1000 CV trials with randomly selected subsets*.

**Figure 4:**
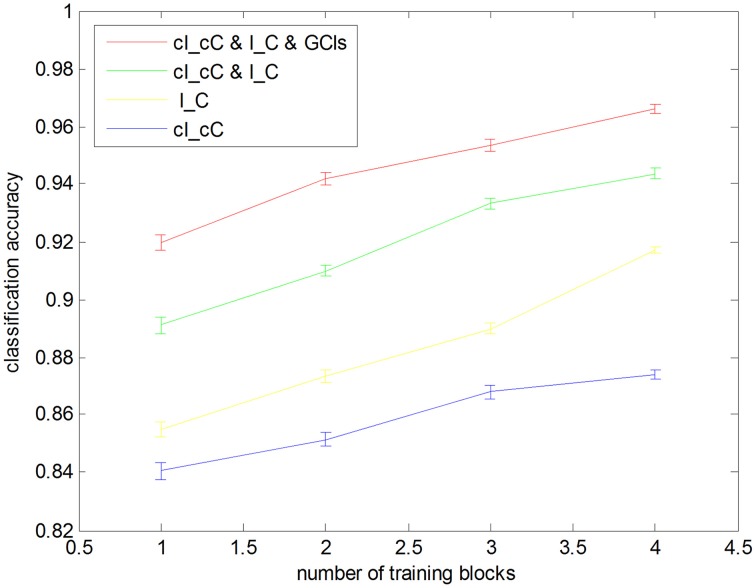
**Leave one block out cross-validation accuracies with different sizes of training blocks**. Each classification accuracy was calculated from averaging 1000 cross-validation trials with randomly selected subsets. Error bars show the standard error.

We also performed CV trials with reduced training blocks using five-fold CV method (Figure 4). In each of the CV trials, we randomly separate the data into five blocks and sampled M (a number ranging from one to four) blocks, which were used for training the classifiers. For each selected *M*, we repeated the sampling and CV 1000 times. Even when we used only one training block, all methods still achieved classification accuracies above chance level (84.06%, the corresponding *p*-value was 0.01).

Classification was also performed using the maximum uncertainty linear discriminant analysis base classifier with the leave-one-out CV (Dai et al., 2012), which achieved a classification accuracy of 96.63% (the corresponding *p*-value was 0.005) with a sensitivity of 87.50% (the corresponding *p*-value was 0.005) and a specificity of 98.00% (the corresponding *p*-value was 0.005) with the activated featured regions from cI_cC, I_C contrasts and GCIs. The feature weights or classification contribution in Tables 3, 4 were obtained by the coefficients of the discrimination hyperplane which measures the contribution of these features to the classification (Dai et al., 2012). As shown, activations in PFC, ACC, Hipp, VTA, insula, and GCIs from IFG to ACC made the main contributions to classification.

## Discussion

This study examined the neural activity and connectivity in response to the performance of an emotional stroop task in adolescents with a family history of SUD (FH+ group) compared to age-matched controls without such a history (FH− group). The fMRI findings reveal consistently strong hyperactivity and reduced connectivity in the FH+ group in numerous brain regions, including PFC, ACC, MCC, insula, hippocampus, putamen, thalamus, STG, and VTA. These brain regions constitute several very important neural circuits, which are thought to be involved in controlling human cognitive behavior in general, and specifically play key roles in decision making, reward and addiction.

Consistent with other behavioral studies of emotional conflict regulation (Etkin et al., 2006, 2010), this study found that FH+ adolescents were unable to adapt to emotional conflict during incongruent trials while conflict adaptation during congruent trials was similar in both FH+ and FH− groups. This demonstrates that the deficit of parental SUD in emotional conflict adaptation and cognitive control may have been transmitted to their adolescents who are substance use-naive.

At the neural circuitry level, previous research has demonstrated that the PFC is responsible for executing cognitive control and producing corresponding adjustments in behavior. ACC controls conflict and emotion, which predicts both greater PFC activity and adjustments in behavior, which appears to play an important role in the engagement of cognitive control (Carter et al., 1998; Botvinick et al., 1999, 2004; Kerns et al., 2004). As in previous stroop fMRI studies (Etkin et al., 2010; Silveri et al., 2011), this study found excessive activation of the PFC and ACC in the FH+ group compared with the FH− group, suggesting that FH+ adolescents might need to engage conflict control related brain regions more actively to compensate for their difficulty processing emotional conflict adaptation. These ideas are consistent with the conflict hypothesis that ACC implements a conflict-monitoring function and then recruits the PFC for execution of cognitive control (Kerns et al., 2004). Moreover, the reduced effective connectivity from PFC to ACC in the FH+ group compared with the FH− group, suggests that uncoupling of the frontal-limbic circuit in FH+ adolescents might be associated with impaired cognitive control.

The FH+ adolescents exhibited greater activity in the insula, putamen, thalamus, STG, and VTA during conflict processing compared with FH− adolescents, indicating the dysfunction of the salience network which unites conflict monitoring, interoceptive-autonomic and reward processing (Seeley et al., 2007; Zielinski et al., 2010). Along with the ACC, these brain areas coactivated in response to varied forms of salience for emotion, homeostatic regulation and reward. The thalamus may help build the bottom-up circuit from basal ganglia to cortex in salience network. When evoking the emotional conflict in the stroop tasks, it might be that FH+ adolescents are more likely to activate brain regions in the frontal-limbic-basal ganglia circuits compared with FH− adolescents. This dysfunction was associated with the cognitive control network in processing conflict and errors (Menon et al., 2001; Kerns et al., 2004; Ridderinkhof et al., 2004), and we therefore speculate that this might be related to the high risk for developing SUD.

Cognitive control, a multifaceted construct defined as the ability to sustain goal directed cognition/behaviors in the face of salient, competing inputs, and its dysfunction, are central factors in the mechanism of SUD risk (Tessner and Hill, 2010). Despite the fact that the sample size here is not a large, the findings provide important insights into the neuroscience of the high risk for developing SUD.

Furthermore, high classification accuracy achieved here suggests that brain activity and connectivity in these regions could truly differentiate the two groups and cross verify the detected differences in activation and connectivity which could be used as biomarkers to distinguish the FH+ group from the FH− group. To our knowledge, this is the first study to show that adolescents who are at elevated risk for SUD by virtue of being FH+ but still substance use naïve, can be accurately classified based on endophenotype markers, which may represent an aspect of an important mechanism of high risk of familial SUD. By identifying such adolescents prior to onset of substance use problems, these findings may be useful in achieving a targeted primary prevention effort, not hitherto available.

Several limitations of the present study should be noted. Firstly, the FH+ and FH− groups should be matched for all other high risk factors for substance use such as socioeconomic deprivation, family history of high risk behavior, childhood history of high risk behavior/conduct disorder/ADHD which are all associated with substance use. Secondly, the sample size in this study was not large. However, we calculated the classification accuracy by averaging 1000 cross validation trials with randomly selected subsets, which cross verify the findings of the abnormalities of brain activity and connectivity in FH+ individuals. The future work should be done on a larger training sample which may lead to higher classification accuracy. In addition, fully independent training and test samples with large sample size will be better for getting a generalizable model (Gabrieli et al., 2015). Finally, emotion and reward systems closely interact (Morrison and Salzman, 2009; Muller-Oehring et al., 2013), higher classification accuracy may be achieved if features are from brain activities of two or more tasks such as MID and emotional stroop task.

In conclusion, the results of the present study reveal that the FH+ adolescents have conflict-related neural hyperactivity and reduced connectivity in the brain regions that are involved in general cognitive control, decision making, and reward system, such as ACC, PFC, and VTA. The high accuracy classification results indicate that these brain features can serve as robust endophenotype makers that distinguish these adolescents who have a positive parental history of SUD and who do not, confirming that the brain circuits which we have detected uncover fundamental differences of neural substrates underlying cognitive behaviors of the adolescents between parental positive and negative histories of SUD, which leads insight into better understanding the endophenotypes for increased risk for developing SUD and developing methods to prevent its familial transmission.

### Conflict of interest statement

The authors declare that the research was conducted in the absence of any commercial or financial relationships that could be construed as a potential conflict of interest.
