# Supplementary material for: Brain activity classifies adolescents with and without a familial history of substance use disorders
Source: Front Hum Neurosci. 2015 Apr 22;9:219. doi: 10.3389/fnhum.2015.00219 (PMC4406072; doi:10.3389/fnhum.2015.00219)
Supplement: Supplementary file 1 [file DataSheet1.DOC]

**SVM Classifier**

In this study, we used SVM to classify the status of family (parental) history of SUD and thus to discriminate between FH+ and FH- adolescents.

Details of the SVM algorithm used to perform the classifications were as follows. Given a training set containing $m$ tuples $\left\{\left(X_{j},y_{j}\right)\right\}_{j=1}^{m}$ where$X_{j}$is the input feature vector which stands for the activity of the ROIs or GCIs, $y_{j}$is the corresponding output which is a FH+ subject or FH- subject. Vectors were first mapped from input space to some feature space by a nonlinear function$z=\varphi\left(x\right)$. Then we separated the different classes by constructing a maximum margin hyperplane.

Mathematically, this involves solving the quadratic programming problem:

$\max\sum_{i=1}^{m} \alpha_{i}-\frac{1}{2}\sum_{i,j=1}^{m} \alpha_{i}\alpha_{j}y_{i}y_{j}k\left(X_{i},X_{j}\right)$ (1)

w.r.t the$\alpha_{i}$is subject to the constraints:

$\sum_{i=1}^{m} \alpha_{i}y_{i}=0, and C\geq\alpha_{i}\geq0, \left(i=1,....,m\right)$ (2)

where$k(X_{i},X_{j})=<\varphi(X_{i}),\varphi(X_{j})>$is the kernel, $\alpha_{i}$are Lagrange multipliers and$C$is regularization parameter. Then the decision function is

$\mathrm{sgn} (\sum_{i=1}^{m} y_{i}\alpha_{i}k(X_{i},X_{j})+b)$ (3)

For example, suppose you have a number of feature vectors $X_{j},j=1,2......J$ which are extracted from the training subject's individual activation map or GCIs. These vectors will then be used as the input of SVM. The output of SVM $y_{j}$is the index of the FH+ participants or FH- controls. Therefore, this is a two-class classification problem. The kernel used in the implementation is the radial basis function (RBF) kernel

$k(X_{i},X_{j})=e^{-\gamma\left\|\left(X_{i}-X_{j}\right)\right\|^{2}}$ (4)

where $\gamma$= 1/ (number of features) is the parameter controlling the width of the Gaussian kernel.

The accuracy of the model was tested by two cross validation methods including ‘Kfold’ and ‘leaveMOut’, which give an estimate of how well the model will generalize to a new data set.
